# Testing and Analysis of Uniaxial Mechanical Fatigue, Charpy Impact Fracture Energy and Microhardness of Two Low-Carbon Steels

**DOI:** 10.3390/ma16020884

**Published:** 2023-01-16

**Authors:** Josip Brnic, Sebastian Balos, Marino Brcic, Miroslav Dramicanin, Sanjin Krscanski, Mladomir Milutinovic, Biao Ding, Zeng Gao

**Affiliations:** 1 Department of Engineering Mechanics, Faculty of Engineering, University of Rijeka, Vukovarska 58, 51000 Rijeka, Croatia; 2 Department of Production Engineering, Faculty of Technical Sciences, University of Novi Sad, Trg Dositeja Obradovica 6, 21000 Novi Sad, Serbia; 3 State Key Laboratory of Advanced Special Steel, School of Materials Science and Engineering, Shanghai University, Shanghai 200072, China; 4 School of Materials Science and Engineering, Henan Polytechnic University, Jiaozuo 454003, China

**Keywords:** uniaxial fatigue and fatigue limit, Charpy impact fracture energy, hardness, C15E+C (1.1141) steel, S235JRC+C (1.0122) steel

## Abstract

The paper presents and analyzes the results of experimental tests performed on two non-alloy low carbon steels (1.1141 and 1.0122) in cases of their exposure to impact fracture energy and uniaxial high cyclic mechanical stress-controlled fatigue. The experimental results provide insight into the changes in the Charpy impact fracture energy of the V-notched test specimen that occur as a result of temperature changes. The experimental results also provide insight into the mechanical response of the tested materials to mechanical uniaxial high-cycle fatigue at room temperature in an air atmosphere and at different applied stress ratios. Material fatigue tests refer to symmetric (R = −1), asymmetric (R = −0.5) and pulsating tensile (R = 0) cycles. The test results are shown in the S–N diagrams and refer to the highest applied stresses in relation to the number of failures at a given stress ratio. Using the modified staircase method, the fatigue limit (endurance limit) was calculated for both tested materials at each prescribed stress ratio. For both tested steel alloys, and at prescribed stress ratios, the fatigue limit levels (σ_f) are shown as follows: for steel C15E+C (1.1141)$\to \sigma_{f}\left[250.8_{R=-1};\;345.4_{R=-0.5};\;527_{R=0}\right]\left(\mathrm{MPa}\right);$ and for steel S235JRC+C (1.0122)→ $\sigma_{f}$[$202_{R=-1};\;310_{R=-0.5};\;462_{R=0}]\left(\mathrm{MPa}\right)$. All uniaxial fatigue tests were performed on unnotched, smooth, highly-polished specimens. The microhardness of both materials was also tested.

## 1. Introduction

A new design of a structure (vehicle, machine, crane, ship, etc.) must have such a level of performance that ensures its economy, durability and safety, which requires efficient use of material and prevention of structural failures [1]. It is to be expected that a structure will be as optimal as possible [2]. All design parameters directly depend on the mechanical response of the structure, i.e., on the properties of the material. Standard design theories prefer structural design against plastic deformation or against fracture. In the first case, the yield strength of the material is considered a criterion against plastic deformation, while in the second case, fracture toughness is considered a criterion against fracture of the structure. Designers, but also users of structures of any kind, try to find a solution to avoid structural failure. Usually, two groups of structural failures can be mentioned—namely, pre-existing material failures, as well as structural failures that may develop over the lifespan of the structure. Commonly-observed modes of mechanical failures in engineering practice can be mentioned, such as fatigue, yielding, corrosion, creep, wear, impact, etc. [3,4,5]. In this paper, the mechanical behavior of structural elements (specimens) made of materials (1.1141 and 1.0122) and subjected to different loads are experimentally examined and analyzed. Mentioned loads (conditions) include uniaxial fatigue of the material due to different stress ratios at room temperature, as well as investigations related to Charpy fracture impact energy that defines the toughness of the material, and finally hardness testing. Furthermore, it should be noted that the results of some mechanical properties of these materials (1.1141 and 1.0122) at room and high temperatures determined based on their engineering stress-strain diagrams, as well as the results of their mechanical creep behavior determined by creep tests, are presented in Refs. [6,7]. Furthermore, designers usually prescribe the level of toughness that a material should possess for a particular application and at a particular temperature. Finally, fatigue of the material is known as mechanical failure mode that can be caused by fluctuations in externally-applied stresses. This is termed mechanical fatigue. When a cyclic (repeated, fluctuated) load is associated with a high temperature, this phenomenon (or failure) is known as creep-fatigue. When the temperature of the cyclic-loaded engineering components also fluctuates, under consideration is thermomechanical fatigue. In addition, when material is subjected to cyclic (repeated) load accompanied with chemically aggressive media, the process is known as corrosion fatigue. It is also known that the fatigue strength of a material, which occurs when the load is repeated, can be significantly lower (reduced) than the strength of the material under a monotonic load. Fatigue of materials whose tests were carried out within the framework of this work belongs to the field of uniaxial fatigue of materials. Otherwise, it is difficult to find research in the literature related to the behavior of these two investigated materials regarding the mentioned environmental conditions. Although both researched materials are quite widespread in technical use, few papers have been published on their behavior in different circumstances, and especially in the areas highlighted here. Some of these papers are listed here. In [8], the influences of low load cycles on fatigue damage in material C15E (1.1141) are investigated in the very-high-cycle fatigue regime. Constant amplitude endurance limits at limiting lifetime of $10^{9}$ cycles are determined in cyclic tension-compression and cyclic torsion tests. Further, in [9], an examination of the behavior of bi-metallic components during the cold upsetting process was made. The researched component consists of a solid inner cylinder around which is fitted a ring of a different material. In one of the experimental studies, a softer C15E core surrounded a ring of mild steel C45E material. Such tests are used to validate the finite-element models. The finite-element method is then used to extend the initial tests to a wider range of cylinder and ring geometries. In [10], the paper focuses on the hot forging of multi-material cladded work pieces using upsetting tests. The research corresponds to gas metal arc welding cladding of an SS316L on a mild steel (C15). A crack mode, specific to clad billets, was observed experimentally and can be predicted by the FE method. Furthermore, in [11], attention is paid to the study of fatigue behavior and damage evolution in cubic low-alloy steels (C15E, C45E, C60E), which contain different ferritic-pearlitic microstructures, and are subjected to high-cycle and very-high-cycle HCF / VHCF regimes. Finally, several published papers relating to material No 1.0122 are mentioned, as follows. In study [12], ${\mathrm{Co}}_{2}B$ nanocrystals, which were synthesized in a planetary type mill, were pre-coated on the surface of S235JRC steel substrate, and the surfaces were then clad. It was found that the hardness and wear resistance of the coatings were approximately three times higher compared to the base metal. In addition, an experimental study was conducted in [13] to determine that a certain value of magnetic flux density of about 230 mT is necessary to enable single-pass laser welding without leakage or melt loss for a combination of austenitic stainless steel AISI 304 and steel S235JRC thickness at an oscillation frequency of about 2.6 kHz. Research dealing with mechanical properties of the C45/S235JR multilayer steel system is presented in [14]. Multilayered steel has been obtained by hot forging and in this way, the system combines the advantages of both materials and reduces the major inconvenience of each material taken separately. In [15], a problem of metal structures corrosion was considered. Since the structure corrosion process runs slowly, for the purpose of corrosion behavior examination, accelerated methods are required. The paper deals with the examination of the corrosion rate of steel rod grades S235JR and S235JRC. The test duration was 42 days and related to a solution of 3.5% NaCl.

The intention (goal) of this work was to experimentally determine the important mechanical properties of these two materials in certain environmental conditions. These properties are relevant for their mechanical behavior in conditions of exploitation, and they enable structural designers to make an easier choice of materials for the design and construction of structures that will work under such conditions during exploitation. In this case, the specified properties that are investigated are material fatigue, impact fracture energy and microhardness. Given that these are materials for the production of engineering structures/building elements in a wide industrial spectrum, especially in the areas of mechanical engineering and construction, knowledge of the properties of these materials plays an important role in their selection and application.

## 2. Basic Data on Experimental Research

Tested materials were two non-alloy, low carbon steels. Both steels (1.1141 and 1.0122), according to the supplier’s certificate, were delivered as a cold-drawn round bar (1.1141, 15 mm in diameter and 1.0122, 18 mm in diameter). Steel 1.1141 is classified as case-hardening steel, while steel 1.0122 is classified as structural steel. Case hardening (or surface hardening) represents a material processing method that is used to increase the hardness in a very thin layer of the outer surface of a metal, and is based on the introduction of carbon into it by diffusion process. However, case-hardening steels are, in fact, structural steels but intended for the surface hardening obtained by a case hardening. The chemical compositions of the tested materials (1.1141 and 1.0122) are quite similar. The geometry and dimensions of the specimens used in testing of both materials are the same, but are significantly smaller than the dimensions of the supplied bars (especially in terms of diameter) from which the specimens are made. The differences in the properties of these two materials are caused by the differences in the diameter of the supplied bars for these two materials. Chemical compositions of these steels are given in Table 1. The tests showed the same results as those previously performed [6,7], when these materials were tested against certain properties. Material (1.1141/C15E+C) is known as unalloyed steel and is usually classified as mild (low carbon, plain carbon) steel. It is engineering steel delivered as cold-drawn round (15 mm) steel bar. It is suitable for the design of parts in many type of engineering needing good wear and fatigue stress resistance. Material (1.0122/S235JRC+C) is a quality steel—or structural steel for the construction industry. It was delivered as cold-drawn round (18 mm) steel bar. Its applications are in civil engineering, bridge building, vehicle industry, mechanical engineering, etc.

Testing Equipment—depending on the type of test performed. The chemical compositions of the tested materials were determined using a GDS500A optical emission spectrometer, LECO (St Joseph, MI, USA) by the smoldering discharge method (argon 99.999%).

Hydraulic pulsator, Schenck Hydropuls PSB (250 kN, Aachen, Germany) was used to test uniaxial material fatigue at room temperature.

Charpy impact machine, Zwick/Roell (300 J, Ulm, Germany) was used to measure the fracture impact energy of the material at different temperatures.

The metallographic investigations regarding hardness were performed by Vickers microhardness tests using a Wilson Tukon 1102 tester, Buehler, IL, USA.

Optical and SEM microscopes were used in microstructure analysis. The microstructure of the considered materials was analyzed using a scanning electron microscope (SEM, JSM6460LV, JEOL, Tokyo, Japan) in secondary electron image (SEI) and backscattered electron image (BEI) mode of operation.

Furthermore, chemical composition of certain phases of interest were determined by 

Energy Dispersive Spectroscopy (EDS). This was made by Oxford Instruments INCA microanalysis system. Previously, specimen cross-sectional samples were coated with gold in a Bal-Tec SCD-005 device. In addition, an optical microscope (OM, Leitz Orthoplan light microscope (LM), Wetzlar, Germany) was used in microstructure analysis of as-received materials.

Test procedures—refer to uniaxial material fatigue tests and to the determination of the Charpy impact fracture energies. Each test procedure is defined by an appropriate standard as set out below.

**Figure 1:**
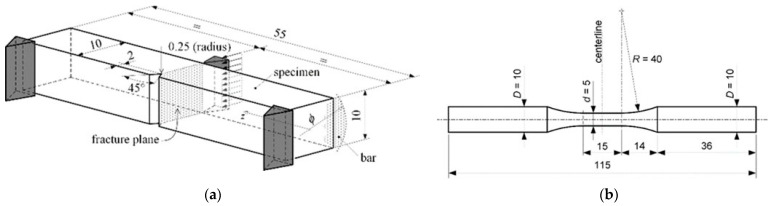
Specimens used in experimental tests (mm): (**a**) Charpy impact test and (**b**) Uniaxial fatigue test.

Specimens—used in material fatigue tests as well as test specimens used in Charpy impact tests, shown in Figure 1. A specific type of test specimen used for a specific test purpose is made in accordance with the standard below. Specimens used in uniaxial fatigue tests were unnotched—smooth, highly-polished specimens. All the samples used in this research were made from rods of the researched materials in such a way that the longitudinal axes of the specimens and bars coincide.

Standards—fatigue uniaxial tests, statistical planning and analysis of data were performed according to the ISO 12107: 2012 (E) standard [16]. Specimens that have been used in uniaxial fatigue tests were manufactured according to the ASTM: E466-96 standard [17]. The standard also prescribes the practice for conducting force-controlled constant amplitude axial fatigue tests of metallic materials.

Charpy tests for notched-bar impact testing of metallic materials were performed according to ASTM E23-12c standard (2015). This standard prescribes the requirements relating to test specimens, test procedures, test reports, etc.

All of the mentioned ASTM standards can also be found in the Annual Book of ASTM Standards (2015) [17].

## 3. Experimental Results and Discussion

### 3.1. Parameters Defining the Strength of the Material

In engineering practice, some significant parameters defining the strength of a material in a certain way are often encountered. These common parameters include ultimate tensile strength, yield strength, creep limit, impact strength, or material fatigue strength corresponding to the certain number of the cycles to the failure, as well as fatigue (endurance) limit. In this paper, impact fracture energy and material fatigue were considered. In addition to these parameters there are also other parameters, such as ultimate tensile strength, yield strength, etc., defining the strength of material in a certain way. A designer must have extensive knowledge and experience of what material properties are required. However, the properties of irons and steels depend on the chemical composition, processing path and resulting microstructure of the material [18]. For a particular steel composition, most properties depend on microstructure and they are known as structure-sensitive properties (for example, yield strength, hardness). In this case, the processing, such as cold rolling, hot rolling, etc., is a means to develop and control the microstructure.

### 3.2. Impact Fracture Testing of a Material Using Charpy Machine

Toughness is considered a measure of the amount of energy required to cause a fracture or failure of the specimen using an impact test. The amount of energy absorbed when the specimen breaks with the impact test (Charpy test) is a measure that gives an indication of the notch toughness of the test material. Test specimens are usually manufactured with a “U” or “V” notch type. In terms of this test, metals can be classified as either brittle or ductile depending on the amount of energy absorbed during the test.

**Figure 2:**
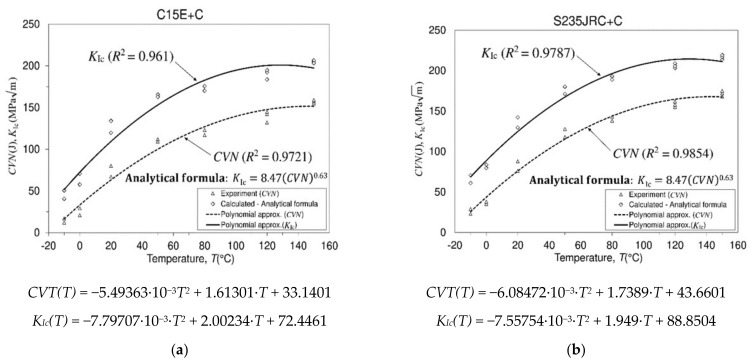
Experimental results of Charpy V-notch impact fracture energy tests and calculated fracture toughness values. (**a**) Material C15E+C (1.1141) and (**b**) Material S235JRC+C (1.0122).

Impact fracture work (impact fracture energy) is measured by experiments carried out on a Charpy impact machine, depending on the test temperature. In this investigation Charpy’s impact fracture energy was determined using V-notched specimens. The diagram representing the dependency of impact fracture energy (CVN) on temperature can have one of the forms shown in in Ref. [19]. Ductile-to-brittle temperature transition (DBTT), shown on the curve representing BCC (body-centered cubic) crystal structure, lies in the transition region representing a point that separates the brittle from the ductile behavior of the material. Impact toughness (generally toughness), also known as impact strength, or threshold of force per unit area before the considered (tested) material undergoes fracture, involves the force application in a very short time (milliseconds). In this sense, the impact toughness represents the amount of energy that the considered material can withstand thanks to the bearing cross-sectional area of the test specimen when a load is suddenly imposed on it. The material will experience fracture or damage if the applied amount of energy exceeds the quantity that it cannot accommodate. General factors affecting toughness are alloying elements, microstructure, service application, strain rate, temperature, stress concentration, strength-to-ductility ratio, fabrication techniques, etc. Metals show low toughness at lower temperatures and experience fracture without significant or with very small plastic deformations (the material is brittle or has a defect). At higher temperatures, toughness increases and significant (large) plastic deformations occur before fracture (the material is tough). Experimentally-obtained results related to Charpy V-notch impact fracture energy of both considered materials (C15E+C and S235JRC+C) are displayed in Figure 2. The same figure also shows the fracture toughness values of both materials, which are actually the calculated values.

For a mild (low carbon, plain carbon) ferrite-pearlite steel with a chemical composition similar to the chemical compositions of the materials investigated in this paper, a diagram of the absorbed impact fracture energy in relation to the temperature in Ref. [20] is shown. The considered materials belong to BCC (body-centered cubic) crystalline structure. The curves representing the dependence of the Charpy impact energy on temperature for different types of steels are shown by graphs in Ref. [15]. Three types of curves are usually displayed. One curve represents the FCC (face-centered cubic) crystal structure, the second the BCC crystal structure, and the third represents brittle materials (high-strength steels). As is known, the most direct difference between BCC and FCC crystal structure is the arrangement of atoms in the crystal. Otherwise, there are also other types of tests that serve to determine some other properties of the material, like the Charpy test, which determines the toughness of a material based on the absorbed impact energy. Each of these properties defines a particular ability of the material relevant to the purpose for which the structure is designed. Despite the existence of numerous material properties, in engineering practice, two of these properties are commonly cited as key mechanical properties of the material, namely, fracture toughness ($K_{\mathrm{Ic}}$) and yield strength ($\sigma_{02})$ [21]. Fracture toughness ($K_{\mathrm{Ic}}$) is usually used as a criterion for the design of the structure against fracture, while the yield strength ($\sigma_{02})$ is usually used for the design of the structure against plastic deformation. A critical value of the stress intensity factor (*SIF* or *K*), designated as $(K_{\mathrm{Ic}}$), determines the material’s resistance against crack propagation, i.e., the material’s resistance to crack extension [22]. We basically distinguish two categories of fracture mechanics in terms of their possibility of application in the analysis of deformation field around the crack tip [23,24,25,26]. The application of linear elastic fracture mechanics (LEFM) may be valid as long as small-scale yielding assumption around the crack tip is valid, while when plastic deformation is present to a great extent, the application of elastic-plastic fracture mechanics (EPFM) is desired. However, due to possible difficulties in its application (complicated manufacturing of specimens due to their geometry, the need for more materials, inability to manufacture them from the real part of the structure, etc.), there are also other types of tests, which provide appropriate measure of fracture resistance. In this sense, the impact test, usually known as the Charpy impact test and widely used in ferrous metals (and plastics), is one such test, as mentioned above. Both Charpy V-notch (*CVN*) impact energy obtained by Charpy test and fracture toughness result obtained by fracture toughness test are parameters reflecting toughness of the material. During numerous tests of fracture toughness ($K_{\mathrm{Ic}}$) and impact fracture energy (*CVN*) of the material, correlations between these two parameters were determined [27,28,29]. The relation that connects the fracture toughness parameter $(K_{\mathrm{Ic}}$) with the impact fracture energy parameter is the well-known Roberts-Newton relation (1) [19,27], which is assumed to be applicable regardless of the test temperature:

(1) $$K_{\mathrm{Ic}}=8.47{\left(CVN\right)}^{0.63},\;[K_{\mathrm{Ic}}\;(\mathrm{MPa}\sqrt{m)}\;;\;\mathrm{CVN}(J)]\;$$

As previously said, the calculated fracture toughness data ($K_{\mathrm{Ic}}$) for both tested materials, based on the impact fracture energy test (*CVN*) data, are shown in Figure 2. Based on the comparison of the diagrams that represent the behavior of the material during the Charpy impact process, it is evident that the behavior has an approximately similar trend and yields similar values at certain temperatures. It can be seen that the differences are not significantly recognizable, and therefore the use of these materials in cases where this property needs to be taken into account meets the set requirements. Considering that Figure 2 shows the experimental data for both tested materials, certain small differences between them are visible, which are the consequences of the dimensions of the cross-sections of delivered rods of test materials and small changes in the chemical composition of these materials. The trend of change in impact energy with temperature change indicates that these are materials with a BCC crystal structure. Furthermore, the approximating curves representing polynomial approximations of the experimental results were chosen in such a way that they approximate the experimental results well (favorably)—that is, they show the trend of impact energy change.

Comparing the obtained data related to Charpy impact energy of the steels considered in this paper with the results of impact energy and assessed fracture toughness of one martensitic steel can be seen with insight in Ref. [30]

### 3.3. Uniaxial Fatigue Testing of Materials

#### 3.3.1. General Consideration

In engineering practice, the term “material fatigue” is widely accepted as a term that refers to changes in the properties of metallic materials that occur in them due to repeated (cyclic, fluctuating) application of stresses (or strains) [31,32]. Cyclic stress can be caused by loads such as compressive, bending, torsional or combined load. The result of the repeated application of stress can occur at a much lower stress level than the fracture stress corresponding to a monotonic load of the same type [25,33]. Two types of fatigue tests are usually mentioned in terms of the number of test cycles, and they are stress-controlled high cycle fatigue (low-level fatigue) and strain-controlled low cycle fatigue (high-level fatigue). The boundary between high cycle fatigue and low cycle fatigue is usually adopted by the number of the cycles in amount of $10^{4}$. The difference between low cycle fatigue (LCF) and high cycle fatigue (HCF) has to do with the deformation. In a case when material fatigue failure occurs under a large number of cycles but stresses and strains remain within the elastic range of the material, the failure mechanism is called high cycle fatigue. In a case when the level of the repeated stress does not belong to the elastic range of the tested material and significant plastic straining occurs, and at the same time, the expected number of the cycles to failure is relatively short, the new type of failure mechanism is under consideration. This failure mechanism is referred to as the strain—controlled (ε−N) or strain-life approach. In addition to these two commonly-mentioned mechanisms that consider the occurrence of fatigue failure, it is worth mentioning the so-called modified Manson-Coffin curve method for estimating fatigue lifetime under complex constant and variable amplitude multiaxial fatigue loading [34].

#### 3.3.2. Uniaxial Mechanical High-Cyclic Fatigue Testing of Materials at Different Stress Ratios

Fatigue of both mentioned materials was tested on smooth, unnotched highly-polished samples at room temperature in the air atmosphere and at different stress ratios, which were R=−1 (fully reversed fatigue), R=−0.5 (asymmetric fatigue) and R=0 (zero-tension fatigue).

In Ref. [35], crack development due to fatigue in a low carbon steel with ferrite-perlite microstructure was investigated. In addition, in Ref. [36], specimens produced from a commercial freight axle made of manganese steel (ingredients: C, Si, Mn, P, S, Fe) were tested to high–cycle fatigue in temperature range (−60 °C to −30 °C) and at room temperature (20 °C), as well as for the Charpy impact test under the same temperature conditions. The stress ratio in fatigue testing was R=−1. The material was normalized and does not contain the constituents such as Cr, Mo, Cu, etc. A medium tensile strength of this material was 590 MPa. For some materials—for example, the previously-mentioned plain carbon steels (middle carbon steels, low carbon steels) and low alloy steels—there is one distinct level of stress below which fatigue failure does not occur under normal fatigue conditions. This stress level is called the fatigue (endurance) limit and in specimens without notches and with a smooth surface finish, is usually marked as $\sigma_{f}$ or $\sigma_{e}$ and is considered as a property of the material [1,31]. In other words, when, regardless of the number of cycles of repeated loading (stress), and under the prescribed conditions of the fatigue test, the test specimen remains unbroken (unfailed), i.e., without the occurrence of a failure, it is said that the stress level, known as fatigue limit, at which the failure does not occur is reached. The main task of experimental tests of material behavior in the fatigue process is to determine the fatigue limit. In engineering practice, for steel materials, instead of an infinite number of cycles during which the material should remain undamaged, the one of 10 million cycles is accepted as a sufficient number of cycles. This was also adopted in this study. A sample that remains undamaged after 10 million cycles is considered to have an infinite lifespan. This assumption is appropriate (economical) but not rigorous. In such a case, the intention is to shorten the time required for testing. The dimensions of the considered engineering element should be determined based on the experimentally-obtained fatigue limit value, i.e., based on the fatigue limit. In addition to the research conducted in this paper, it is interesting to get acquainted with some other research in this area. In this regard, in Ref. [37], fatigue evaluation and CFRP strengthening of diaphragm cutouts in orthotropic steel decks were performed. Additionally, in Ref. [38], fatigue property analysis of U-rib-to-crossbeam connections under heavy traffic vehicle load was made, while in Ref. [39], the properties of steel in the area of fatigue and creep were investigated.

#### 3.3.3. Stress Life (*S* − *N*) Diagram and Fatigue Limit Calculation

**Figure 3:**
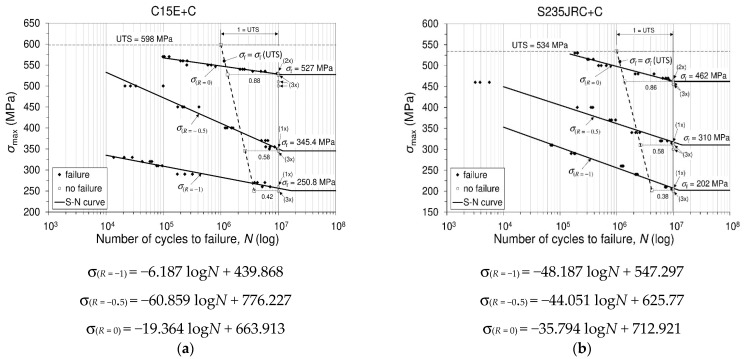
Stress-life (*S-N*) diagrams at different stress ratios (C15E+C; S235JRC+C). (**a**) Material C15E+C (1.1141) and (**b**) Material S235JRC+C.

In accordance with the desired goal of determining the fatigue (endurance) limit of the material, uniaxial high-cycle mechanical fatigue tests of sinusoidal shape stress cycle were performed in this study for each of the tested materials and the prescribed stress ratios. The fatigue tests were performed in a decreasing stress regime for each of the prescribed stress ratios. For each stress level applied, at the prescribed stress ratio, the fatigue test of the material leads either to failure (fracture) of the sample, or the sample remains unbroken after 10 million cycles ($10^{7}$). In this testing, a test that did not cause the specimen to fracture is considered a test in which the specimen has withstood more than $10^{7}$ cycles, which is commonly adopted in engineering practice for steel alloys. The fatigue stress-life (S−N) diagram (plot), or (S−N) curve, was constructed based on material fatigue testing according to the stress-life model, which was performed in accordance with the ISO standard [17]. On the ordinate of the mentioned (S−N) diagram, the levels of maximum applied stresses are plotted, and on the abscissa, the number of cycles to the failure (fracture) of the specimen are plotted. Each test performed results in either a fracture of the specimen (♦), or the specimen remains unbroken (○), which is entered into the coordinate system of the diagram as a single point (mark). In Figure 3, fatigue stress-life diagrams related to C15E+C and S235JRC+C steels are shown. For both materials, tabular data are given, which refer to the applied stresses, the number of failed and unfailed specimens and constants, based upon which the values (levels) of fatigue (endurance) limits for each tested material are calculated. It should be noted that the S-N diagram, obtained on the basis of experimental testing, consists essentially of two parts. The first part (area) of the S-N diagram belongs to the so-called finite fatigue region (finite fatigue life), while the second part belongs to the so-called infinite fatigue region (infinite fatigue life). In this sense, the first part (finite fatigue region) may be represented by an inclined line if measured data are approximated by logarithmic line, while the second part (infinite fatigue region) of the diagram may be represented practically by a horizontal line. This is usually true for test material that shows a clear limit to fatigue; otherwise, the *S-N* curve may possess a shape that corresponds to material without a clearly-defined fatigue limit.

The following is a tabular presentation of the data needed to calculate the fatigue limit of each of the tested materials.

Experimental tests of both investigated materials were carried out in accordance with the data given in Table 2, Table 3 and Table 4, considering both materials. Furthermore, according to these data, and according to the formulae given in Table 5, the fatigue limits of both materials were calculated for the predicted stress ratios. Finally, a complete insight into the fatigue behavior of both materials, as well as the display of fatigue limits of both materials, is given in Figure 3. In addition, numerically calculated values required to determine the fatigue limits of the tested materials, as well as calculated values of the fatigue limits of the materials, are shown in Table 6. This Section 3.3.3 presents the results of the behavior of two chemically similar materials, with respect to uniaxial high-cyclic mechanical fatigue. The applied high-cyclic repeating uniaxial stresses in both materials were performed under the same stress ratios. As can be seen from the stress-life diagrams of both materials, there are certain differences in the fatigue strength levels of the tested materials at the same ratio of applied stresses and at the same number of fatigue cycles (Figure 3). Depending on the stress ratio and the magnitude of the maximum stress, fatigue tests were conducted at approximately 30 Hz. The difference in the levels of fatigue strength obtained at the same considered number of stress cycles of these two materials is consistent with the difference in their monotonic tensile strength, which results due to the difference in the cross-sectional dimensions of the delivered materials and small differences in their chemical composition. The difference in the level of fatigue limit can be considered in the same way. Since the “fatigue limit” levels are determined by the ISO standard using the modified staircase method, the obtained results of the ”fatigue limit” provide structural designers with the security of using the selected material at a defined number of cycles without fracture or failure occurring (the so-called infinite fatigue region). Based on the presented experimental results in Figure 3, as well as the calculated values of fatigue limit for each material tested, it is possible to determine the following:

- the ratio of the fatigue limit to the monotonic tensile strength of the material for a particular stress ratio at which the fatigue test was conducted;
- an increase in the fatigue limit of an individual material with an increase in the stress ratio for an individual material, as well as an increase in the ratio between the fatigue limit and monotonic tensile strength, as stress ratio increases; and
- relationship of the levels of fatigue limits obtained for the tested materials at certain stress ratios.

It can be seen that the ratio of the fatigue limit to tensile monotonic strength at the same stress ratios for both materials are quite similar. For a simpler presentation, in Table 6, the results are shown in the form of integers. In fact, the decimal values are rounded to the value of integers, which does not disturb either the individual absolute value or the mutual ratios of the results. In Ref. [40], an analysis is presented regarding the lifetime multi-hazard fragility evaluation by considering wind-induced fatigue. In addition, consideration of the *S-N* curves in the high-cycle fatigue regime, regarding component design and safety, can be found in Ref. [41].

Remark: If in Table 2, Table 3 and Table 4, in the columns named “specimen”, then in the columns named “failed” as well as in the columns under the label “R” (stress ratio), only one mark or only one number appears, it means that the value is the same for both considered materials. If two marks or two numbers appear in the mentioned columns, then the second mark or the second number refers to the material S235JRC+C. In the following, the fatigue limit for the investigated materials was calculated using formula (2), and the necessary elements included in this calculation were determined using formulas (3) and (4) given in Table 5.

The model used for fatigue testing and determination of fatigue strength (as well as “fatigue limit”) has certain limitations, but is very suitable for engineering practice. On the other hand, the scatter analysis method can be considered useful when there are a small number of data points. However, if there are many points to plot, then the scatter plot becomes cluttered and unreadable. Furthermore, it only shows the direction, and not the degree of correlation, between two variables.

### 3.4. Microhardness Testing

**Figure 4:**
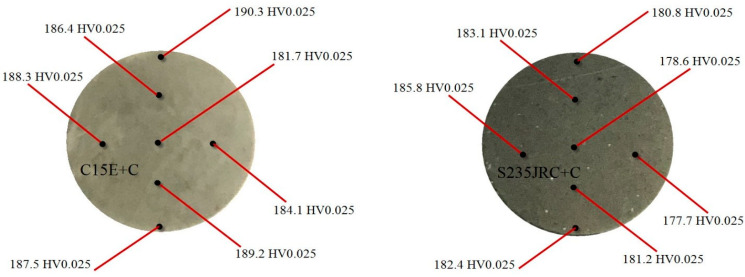
Microhardness test: C15E+C (left) and S235JRC+C (right) steel alloys.

The hardness of the material is a relevant mechanical property of the material that belongs to a type of structure-sensitive properties. Hardness is usually defined as a measure of the resistance of a material to its localized plastic deformation which can be induced by mechanical indentation or abrasion. Due to the complex geometry of the samples used in testing other material properties, and based on the linear correlation between hardness and tensile strength of the material, hardness is often used in assessing the ability of a given component to survive and perform its function. Using the Vickers test, hardness calculation is independent of the size of the indenter. The indenter must have high resistance to self-deformation. The basic principle is to determine the material’s ability to resist plastic deformation caused by its source/exciter. The basic unit of Vickers hardness is the hardness number (HV), and this can be transformed into Pascal units. The hardness number represents the ratio of the applied force to the diamond (indenter) and the surface area of the resulting indentation (not on the surface normal to the force), and is therefore not the pressure. Microhardness is to be considered the hardness of a material determined by indenter indentation using low loads. Imprints are usually very small so they must be examined under a microscope. The term microhardness is often misinterpreted as “very low hardness”. Microhardness (HV0.025) of the ferrite phase was measured with 25 g loading (corresponding to a force of 0.2452 N) at different locations along the cross-section of polished samples (as-received condition), and average values were calculated for each material. The results of the microhardness testing are shown in Figure 4. As can be seen, the measured microhardness values are in the range of 181.7–190.3.2 HV0.025 (on average, 186.8 HV0.025) for C15E+C material, while for S235JRC+C material they range between 177.3 and 185.8 HV0.025 (on average, 181.4 HV0.025). It could therefore be said that there is no significant difference in the hardness between two analyzed materials. Slightly higher microhardness values in the case of C15E+C material can be attributed to differences in microstructure between the two materials. Namely, compared to S235JRC+C material, C15E+C material has a finer microstructure with smaller grain size. As for the uniformity of microhardness, distribution differences are negligible, i.e., distribution is highly uniform. For both materials, deviations in microhardness from the average values are less than 5%.

### 3.5. Microstructure Analysis of Tested Materials

**Figure 5:**
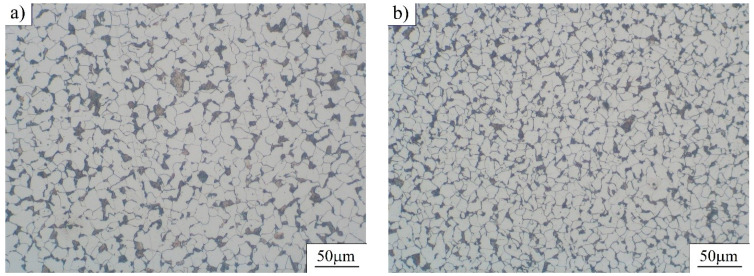
Microstructure of tested steels (as-received materials)—images taken using optical (LM) microscope: (**a**) S235JRC+C steel and (**b**) C15E+C steel.

**Figure 6:**
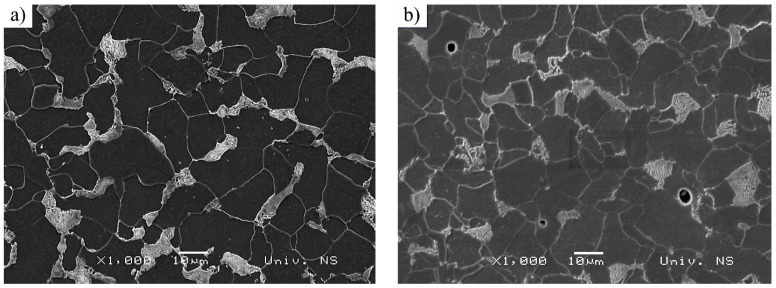
Microstructure of tested steels (as-received materials)—images taken using SEM microscope: (**a**) S235JRC+C steel and (**b**) C15E+C steel.

However, metallographic tests related to as-received C15E+C and S235JTC+C materials were performed on prepared cross-sections using optical microscope, as mentioned below. The tests related to the fracture surfaces of the considered materials subjected to uniaxial mechanical fatigue, and were performed on both materials under the same fatigue conditions (stress ratio and magnitudes of applied stress were: (R=−0.5;−220 MPa/400 MPa). C15E+C material was failed at 1,527,527 cycles, while S235JRC+C material was failed at 352.348 cycles. Cross-sections of the specimens were prepared by a standard metallographic preparation procedure: specimen cutting, mounting, grinding (100 to 3000 grit SiC abrasive papers) and polishing (6; 3; 1 and ¼ µm diamond suspensions). Etching was done by Nital (2 % nitric acid-HNO_3_ in alcohol). Etched microstructures of two tested materials obtained by optical (LM) and SEM microscopes are shown in Figure 5 and Figure 6. It can be seen that the microstructure consists of ferrite and pearlite, which is in accordance with the chemical compositions of the considered materials. C15E+C steel possesses a slightly finer microstructure—that is, polygonal ferritic grains. Pearlite is of lamellar type.

### 3.6. Fracture Surfaces

Both the considered steels were subjected to the same loading conditions as mentioned above (R=−0.5;(−220 MPa/400 Mpa). Number of cycles to failure (fracture) was: 352.348 cycles (S235JRC+C); 1.527.527 cycles (C15E+C).

**Figure 7:**
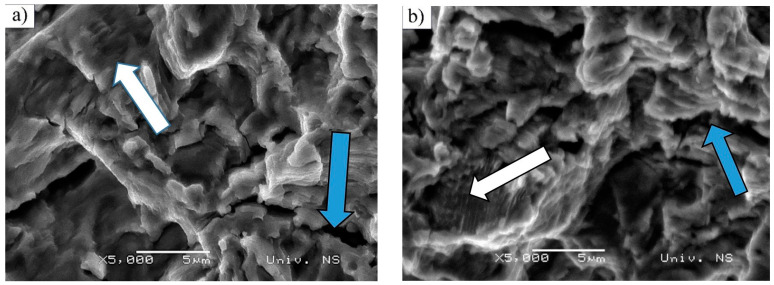
Fatigue crack propagation zone of tested steels—image taken using SEM microscope: (**a**) S235JRC+C steel and (**b**) C15E+C steel.

**Figure 8:**
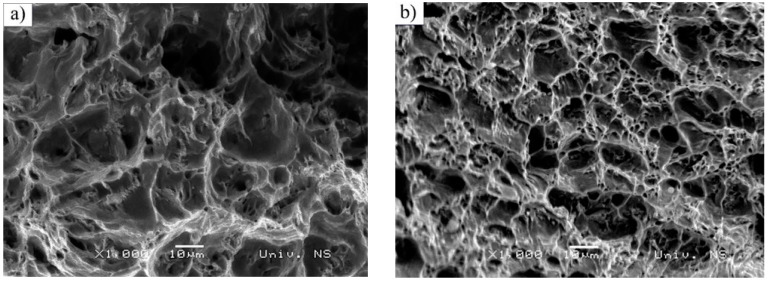
Final fracture zone in tested steels-image taken using SEM microscope: (**a**) S235JRC+C steel and (**b**) C15E+C steel.

Fatigue crack propagation zones and final fracture zone surfaces are shown in Figure 7 and Figure 8, respectively. In Figure 7, fatigue crack propagation zones are presented for both S235JRC+C (Figure 7a) and C15E+C (Figure 7b) steels. Fatigue striations can be observed in both materials (white arrows). They represent incremental growth of the fatigue crack, forming the multi-faceted propagation surface, which can be found in polycrystalline materials and transgranular fractures. The most active slip planes dictate the fracture itself [42]. Other slip planes can be seen in the form of secondary cracks within the fracture surface (blue arrows). As could be observed in Figure 5 and Figure 6, facets are finer in C15E+C steel, which is in accordance with the slightly finer ferritic grains in this material. The final fracture zone in S235JRC+C steel and in C15E+C steel is shown in Figure 8.

These fracture surfaces are dimpled, showing the ductile nature of both materials. It is clear the dimples are significantly coarser in S235JRC+C steel compared to the ones in the fracture surface of C15E+C steel, which is also an indicator of finer microstructure in C15E+C material, apparent in Figure 5, Figure 6 and Figure 7.

Fatigue conditions: R=−0.5;(−200 MPa/400 Mpa). Number of cycles to failure: 1.527.527 (C15E+C); 352.348(S235JRC+C).

Fatigue conditions: R=−0.5: (−200 MPa/400 Mpa). Number of cycles to failure: 1.527.527 (C15E+C); 352.348 (S235JRC+C).

**Figure 9:**
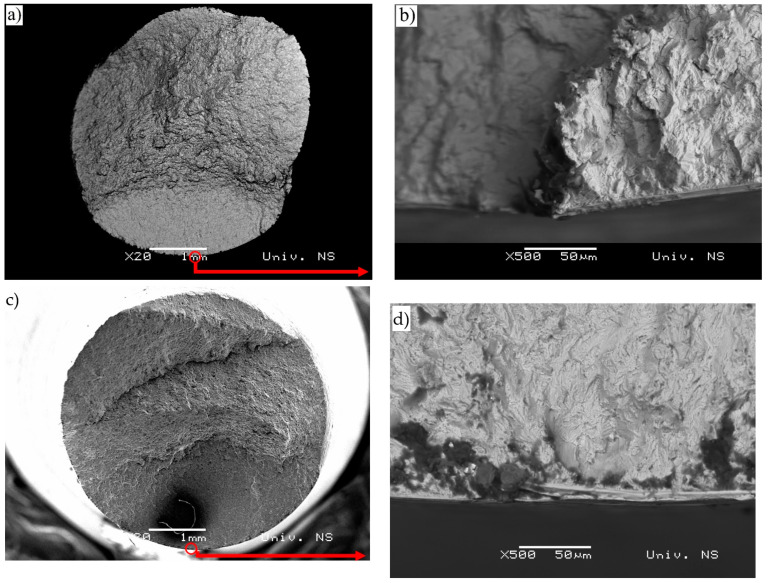
S235JRC+C and C15E+C steel alloys: macro images, inclusions and crack sites: (**a**) S235JRC+C: Macro image-specimen fracture surface, (**b**) Inclusions in undersurface area and crack site, (**c**) C15E+C: Macro image-specimen fracture surface and (**d**) Inclusions in undersurface area and crack site.

Macro images of fracture surface and details of crack initiation site in S235JRC+C steel are presented in Figure 9a,b. Crack initiation occurred in the bottom of the specimen fracture surface (Figure 9a—marked with a circle) and presented in backscatter electron mode (Figure 9b), with indication of the presence of undersurface inclusions. The situation is similar with material C15E+C (Figure 9c,d). Fatigue conditions are as previously defined in Figure 7 and Figure 8 (given above).

### 3.7. EDS Analysis

**Figure 10:**
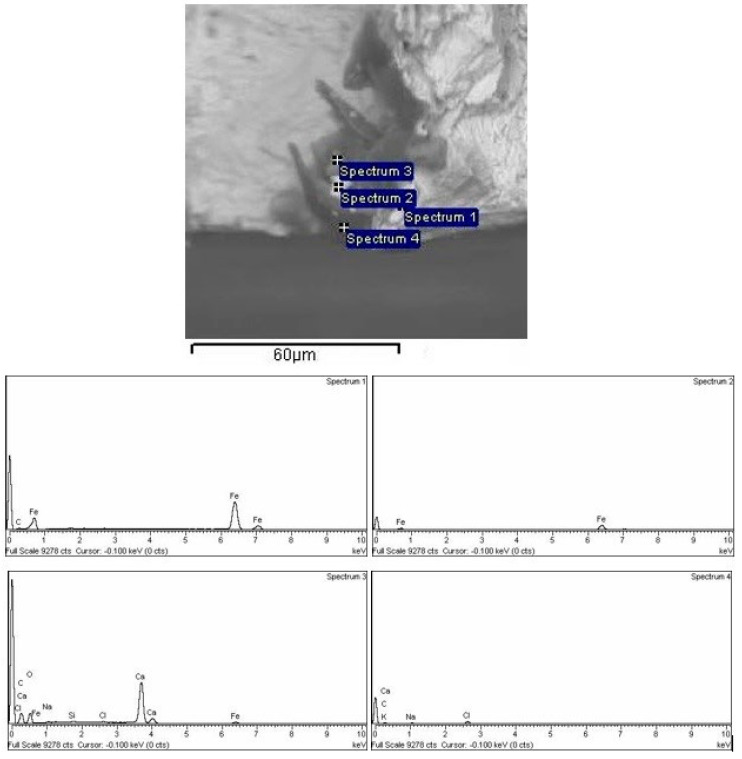
EDS analysis of S235JRC+C: nonmetallic inclusion.

**Figure 11:**
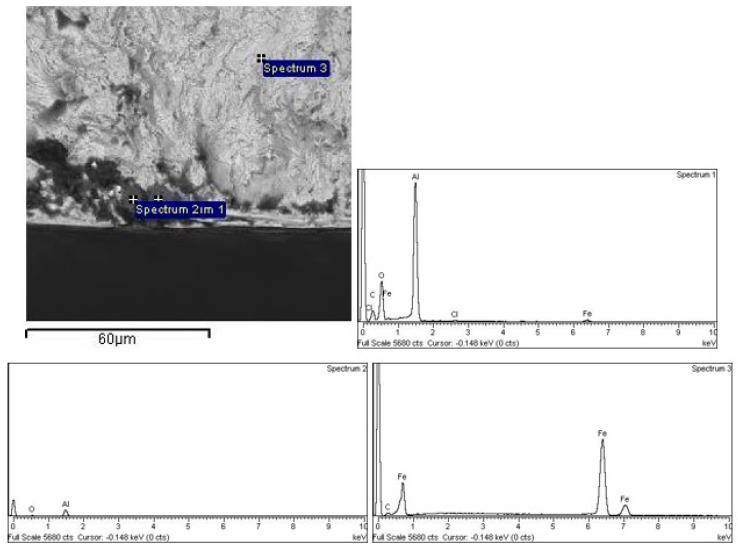
EDS analysis of C15E+C: nonmetallic inclusion.

The results of energy dispersive spectroscopy (EDS) analysis are shown in Figure 10 and Figure 11. In Figure 10, the BEI image of the S235JRC+C steel surface was analyzed in the apparently darker area with a different chemical composition to the matrix. It can be seen that inclusions in S235JRC+C (Spectra 3 and 4) correspond to calcium-based impurities, while other spectra refer to base metal, Figure 10. Ca is added in order to avoid the formation of coarse, clustered alumina inclusions and form smaller compact spherical inclusions [43]. In C15E+C, subsurface inclusions closely correspond to Al_2_O_3_ inclusions, Figure 11.

Table 7 shows the types of testing, test results and possible damage.

## 4. Conclusions

The study presents experimental results and behavior analysis of two non-alloy low carbon steels (C15E+C and S235JRC+C) subjected to Charpy (V-notch) impact fracture tests and uniaxial high-cycle mechanical fatigue tests. The details that characterize the test results are given as follows:

- Both investigated materials were tested for Charpy V-notch impact fracture energy in the temperature range from (−10 °C) to (+150 °C). For both tested materials, experimental results on the absorbed impact fracture energy as a function of test temperature are presented. Based on these data, curves were plotted that approximately outline the trend of absorbed fracture energy due to temperature change. These approximate curves simultaneously indicate that both considered materials belong to the BCC crystal structure. For this structure, if the unit cell edge length is “a” and the atomic radius “R”, then the measure “a =$\;4R/\sqrt{3}$.”
- Both investigated materials were tested for uniaxial high-cyclic mechanical fatigue at room temperature and at several stress ratios (R=−1; R=−0.5; R=0). For each of the tested materials, a stress-life (S-N) diagram is shown and the fatigue limit is calculated using the modified staircase method.
- The microhardness of both materials was tested and referenced in the text of the paper.

## 5. Summaries

- The average measured impact fracture energy in C15E+C steel, for example, at these two temperatures, is (−10 °C→14.5 J and +150 °C→157 J), respectively, and in the case of S235JRC+C steel, it is (−10 °C→26 J and +150 °C→171.6 J), respectively. The behavior of both considered materials is similar, and the obtained values are also slightly different.
- For each of the tested materials at each stress ratio (R), the fatigue limit ($\sigma_{f}$) as a function of ultimate tensile strength (designated as $\sigma_{m}\;or\;UTS)$ is defined in the form $\sigma_{f\left(R\right)}/UTS=x.$ In this sense, for both tested materials the following results are obtained:
  $$C15E+C\;\mathrm{steel}:\;[250.8_{R=-1}/598=0.42;\;345.4_{R=-0.5}/598=0.58;\;527_{R=0}/598=0.88]$$
  $$S235\mathrm{JRC}+C\mathrm{steel}:\;[202_{R=-1}/534=0.38;310_{R=-0.5}/534=0.58;\;462_{R=0}/534=0.86]\;$$
- The average value of microhardness is:
  C15E+C: 186.8 HV0.025; S235JRC+C: 181.4 HV0.025

## Figures and Tables

**Table 1:** Chemical composition (wt %) of the investigated materials (Fe is balance).

Chemical Element	C	Cr	Si	Mn	P	S	Mo	Cu	Al	W	Sn	Nb	Co
Mat. 1.1141	0.135	0.084	0.233	0.38	0.01	0.009	0.019	0.027	0.035	0.006	0.007	-	-
Mat. 1.0122	0.162	0.158	0.237	0.534	0.011	0.012	0.009	0.039	0.027	0.003	0.008	0.003	0.002
Mat. 1.1141	Fe	98.995											
Mat. 1.0122	Fe	98.767											

**Table 2:** Data for modified staircase method.

Stress Ratio (R): (A) R=−1; (B) R=−0.5; (C) R=0 Room Temp., Specimen Failed(◆), Specimen Unfailed (○)								
Stress Ratio $R=\frac{\sigma_{min}}{\sigma_{max}}$$\left[\frac{\mathrm{MPa}}{\mathrm{MPa}}\right]$		Specimen						
		1	2	3	4	5	6	7
**C15E+C** */S235JRC+C*								
(A) R=−1								
**−270/270**	*−210/210*			♦		♦		♦
**−260/260**	*−205/205*		♦/○		♦		○	
**−250/250**	*−200/200*	○						
(B) R=−0.5								
**−175/350**	*−160/320*			♦		♦		♦
**−172.5/345**	*−157.5/315*		○		♦		♦	
**−170/340**	*−155/310*	○						
(C) R=0								
**0/535**	*0/470*			♦		♦		♦
**0/530**	*0/465*		○		♦		○	
**0/525**	*0/460*	○						

**Table 3:** Data analysis related to Table 2.

Stress Ratio(R): (A) R=−1;(B) R=−0.5; (C) R=0 Room Temp., f-Specimen Failed					
Stress Ratio $R=\frac{\sigma_{min}}{\sigma_{max}}$$\left[\frac{\mathrm{MPa}}{\mathrm{MPa}}\right]$		Stress Level, i	*f*_i_	*if*_i_	*i* ^2^ *f*_i_
			f−Failed		
**C15E+C**/*S235JRC+C*					
(A) R=−1					
−**270/270**	*−210/210*	**2**	**3**	**6**	**12**
−**260/260**	*−205/205*	**1**	**2**/*1*	**2**/*1*	**2**/*1*
−**250/250**	*−200/200*	**0**	**0**	**0**	**0**
$\sum^{}f_{i},\;if_{i},\;i^{2}f_{i}$			**5**/*4*	**8**/*7*	**14**/*13*
(B) R=−0.5					
−**175/350**	*−160/320*	**2**	**3**	**6**	**12**
−**172.5/345**	*−157.5/315*	**1**	**2**	**2**	**2**
−**170/340**	*−155/310*	**0**	**0**	**0**	**0**
$\sum^{}f_{i},\;if_{i},\;i^{2}f_{i}$			**5**	**8**	**14**
(C) R=0					
**0/535**	*0/470*	**2**	**3**	**6**	**12**
**0/530**	*0/465*	**1**	**1**	**1**	**1**
**0/525**	*0/460*	**0**	**0**	**0**	**0**
$\sum^{}f_{i},\;if_{i},\;i^{2}f_{i}$			**4**	**7**	**13**

**Table 4:** A, B, C and D constants, calculated (ISO standard [17]).

Stress Ratio (R ); Constants			
Formula	R=−1	R=−0.5	R=0
C15E+C*/S235JRC+C*			
$A=\sum^{}i\cdot f_{i}$	**8**/7	**8**	**7**
$B=\sum^{}i^{2}\cdot f_{i}$	**14**/13	**14**	**13**
C=¾	**3**/4	**5**	**4**
$D=\frac{B\cdot C-A^{2}}{C^{2}}$	**0.24**/0.1875	**0.24**	**0.1875**

**Table 5:** Fatigue limit calculation procedure.

The Value To Be Determined	Formula (Equation) ISO Standard [17]	Equation Number
Fatigue limit $\left(\sigma_{f}\right)$	$\sigma_{f\left(P,\;1-\alpha \right)}={\bar{\mu }}_{y}-k_{\left(P,1-\alpha ,\right)}\cdot {\bar{\sigma }}_{y},$	(2)
Mean fatigue strength $\left({\bar{\mu }}_{y}\right)$	${\bar{\mu }}_{y}=\sigma_{0}+d\left(\frac{A}{C}-\frac{1}{2}\right)$	(3)
Estimated standard deviation of the fatigue strength (${\bar{\sigma }}_{y})$	${\bar{\sigma }}_{y}=1.62\cdot d\left(D+0.029\right)$	(4)
In Equations (2)–(4) there are:	$k_{\left(P,1-\alpha ,\nu \right)}$, the coefficient for the one-sided tolerance limit for a normal distribution.. $\sigma_{0}$ is the lowest stress level, see Table 3. “d” is the stress step (the difference between the neighboring stress levels), seeTable 3. “A, C and D”, see Table 4.	-
Items: ν, n, P, (1−α)	In accordance with the recommendation of the mentioned ISO standard, the value ν=n−1=6, where n is the number of items in a considered group. In addition, if a desired probability is P=10%, and a confidence level (1−α) = 90%, according to the table B1, given in ISO standard [17], it is:$\;k_{\left(P,1-\alpha ,\nu \right)}\;$= $k_{\left(0.1;0.9;6\right)}=$2.333	-

**Table 6:** Calculated values based on fatigue test results and Table 5.

Materials									
C15E+C UTS = 598 MPa [6]			S235JRC+C UTS = 534 MPa [7]						
Used Table 2, Table 3 and Table 4									
		Stress Ratio, R							
Calculated value, Table 5	Equation Number	−1		−0.5	0	−1		−0.5	0
		Values (MPa)							
Mean fatigue strength $\left({\bar{\mu }}_{y}\right)$	3	261		346	531	206		316	466
Estimated standard deviation (${\bar{\sigma }}_{y})$	4	4.36		1.75	1.75	1.75		2.18	1.75
Fatigue limit ($\sigma_{f})$	2	250.8		345.4	527	202		310	462
Ratio: $\sigma_{f}/UTS$		0.42		0.58	0.88	0.38		0.58	0.86

**Table 7:** Investigated materials: Load, testing, test results and possible damage.

Type of Load	Type of Testing	Possible Failure (Damage)	Test Result
Repeated (cyclic) load	Uniaxial mechanical fatigue testing	Fracture of engineering component	Fatigue strength Fatigue limit (room temp.)
Impact load	Charpy V-notch impact testing	Fracture (separation) of considered element	Impact fracture energy (different temp.)
Applied load by diamond pyramid (indenter)	Vickers microhardness testing	Plastic deformation	Average Vickers microhardness values(room temp.)

## Data Availability

Not applicable.
